# Body mass index and the risk of basal cell carcinoma: evidence from Mendelian randomization analysis

**DOI:** 10.7717/peerj.14781

**Published:** 2023-01-24

**Authors:** Likui Lu, Bangbei Wan, Hongtao Zeng, Jun Guo, Min Li, Miao Sun

**Affiliations:** 1 Institute for Fetology, The First Affiliated Hospital of Soochow University, Suzhou, Jiangsu, China; 2 Reproductive Medical Center, Hainan Women and Children’s Medical Center, Haikou, Hainan, China; 3 Department of Urology, Central South University Xiangya School of Medicine Affiliated Haikou Hospital, Haikou, Hainan, China; 4 Department of Dermatology, The First Affiliated Hospital of Soochow University, Suzhou, Jiangsu, China; 5 Department of Dermatology, Dushu Lake Hospital Affiliated to Soochow University, Suzhou, Jiangsu Province, China; 6 Dushu Lake Hospital Affiliated to Soochow University, Suzhou, Jiangsu Province, China

**Keywords:** Basal cell carcinoma, Body mass index, Mendelian randomization, Genome-wide association study, Single-nucleotide polymorphisms

## Abstract

**Objective:**

We aim to test whether body mass index (BMI) is causally associated with the risk of basal cell carcinoma (BCC) using Mendelian randomization (MR) analysis.

**Methods:**

Single-nucleotide polymorphisms (SNPs) associated with four BMI-related traits were screened *via* a genome-wide association study (GWAS) with 681,275, 336,107, 454,884, and 461,460 European-descent individuals, respectively. Summary-level data for BCC (17,416 cases and 375,455 controls) were extracted from UK Biobank. An inverse variance weighted (IVW) method was employed as the primary MR analysis. Sensitivity analyses were conducted *via* MR-Egger regression, heterogeneity test, pleiotropy test, and leave-one-out sensitivity test. The assumption that exposure causes outcome was verified using the MR Steiger test. Meta-analysis was also used to estimate the average genetically predicted effect of BMI on BCC.

**Results:**

Two-sample MR analysis showed inverse associations between genetically predicted BMI and BCC risk. Moreover, when exposure and outcome were switched to see if reverse causation was possible, there was no evidence of a cause-and-effect relationship from BCC to BMI. Finally, the meta-analysis also showed a strong negative causal relationship between BMI and BCC.

**Conclusion:**

Genetical predicted higher BMI were associated with lower BCC risk. Further research is required to comprehend the mechanisms underlying this putative causative association.

## Introduction

Skin cancer is one of the most common malignancies in the world and is an important public health concern. Malignant nonmelanoma skin cancers originate from keratinized epithelial cells, and these cancers include basal cell carcinoma (BCC) and squamous cell carcinoma (SCC). Melanoma only accounts for about 2% of malignant skin cancer (Agbai et al., 2014). However, BCC, formerly referred to as basal cell epithelioma, is the most common form of skin cancer and most prevalent cancer in humans, particularly in elderly populations (Garcovich et al., 2017; Marzuka & Book, 2015). Age and sun exposure are the most significant and well-established risk factors for BCC in paler/white skin (van der Poort et al., 2020). Additional risk factors include ionizing radiation, chronic inflammatory skin diseases, and exposure to arsenic (Pellegrini et al., 2017; Surdu et al., 2013).

Obesity has been connected with a variety of cancer types in humans. In the context of skin cancer, obesity has been found to be positively related to melanoma in males but not in women (Dobbins, Decorby & Choi, 2013; Sergentanis et al., 2013). Similarly, epidemiologic evidence addressing the association between body mass index (BMI) and BCC was contradictory, with several studies suggesting an inverse relationship (Zhang et al., 2017; Zhou, Wu & Luo, 2016), and other studies demonstrated that there was no association between BMI and BCC risk (Milan et al., 2003; Olsen et al., 2006). The causes of the variability between these studies are unknown, and it remains to be determined whether there was a dose–response relationship or whether such an inverse relationship was simply explained by confounding factors of sun exposure and sensitivity assessments.

A relationship between being overweight and skin cancer may have both biological and behavioral origins. Biologically, a higher BMI is connected with increased circulating estrogen levels (De Pergola & Silvestris, 2013), which in mice models have been linked to a decreased incidence of BCC (Mancuso et al., 2009). From a behavioral perspective, ultraviolet radiation (UVR) exposure is a major risk factor for skin cancer, and differences in outdoor activity levels due to BMI (Tang et al., 2013; Wareham, Van Sluijs & Ekelund, 2005) may contribute to differences in UVR exposure. As a result, exposures to UVR and/or estrogen may serve as potential mediators of the connection between BMI and skin cancer. However, this conclusion may not always be true. Previous observational study conducted by Zhang et al. (2017) reported that BMI was negatively related to early-onset BCC (OR = 0.43, 95% CI [0.26–0.71]), and neither UVR nor estrogen-related exposures in women could account for this connection. Although useful, these observational studies are prone to confounding influences, resulting in incorrect causal findings. As a result, randomized controlled trials are required to determine whether the associations discovered in observational research are valid.

Randomized studies on skin cancer are complex because large sample sizes and prolonged follow-up are required. Therefore, drawing the causal connection between BMI and BCC becomes challenging. In order to determine whether or not the stated association between BMI and BCC is a causal relationship, an effective method need to be utilized. Mendelian randomization (MR) research has the potential to address this question in particular (Do et al., 2013; Emdin, Khera & Kathiresan, 2017; Frikke-Schmidt et al., 2008; Voight et al., 2012). Randomizing alleles during gamete production equalizes confounding factors, similar to a randomized trial. Furthermore, genetic variation can affect outcomes, but outcomes cannot affect genes, so no inference of reverse causality can be drawn. MR assumes the genetic variant is related with the exposure, not confounders, and impacts the result only through the exposure (Emdin, Khera & Kathiresan, 2017). Causality can be established through a genetic instrument that fits all MR assumptions. Previous research using MR approaches successfully predicted the causative effect of BMI on many cancers, including lung cancer (Zhou et al., 2021), colorectal cancer (Suzuki et al., 2021), and melanoma (Dusingize et al., 2020).

However, to our knowledge, there are currently no reports on the relationship between BMI and BCC using MR methods. Thus, we conducted a two-sample MR analysis and meta-analysis to investigate the causal relationship between BMI and BCC, utilizing the summary data from the largest BMI and BCC GWASs to date.

## Methods

### Mendelian randomization

#### Study design

The summary-level data used in the two-sample MR analysis came from the IEU Open GWAS database (https://gwas.mrcieu.ac.uk/), including four BMI datasets (GWAS ID: ieu-b-40, 681,275 individuals of European descent; ukb-a-248, 336,107 individuals of European descent; ukb-b-2303, 454,884 individuals of European descent; ukb-b-19953, 461,460 individuals of European descent) and one BCC dataset. The relevant ethics committee authorized initial GWAS, and all subjects supplied informed permission.

### Assumptions of mendelian randomization study

**Figure 1:**
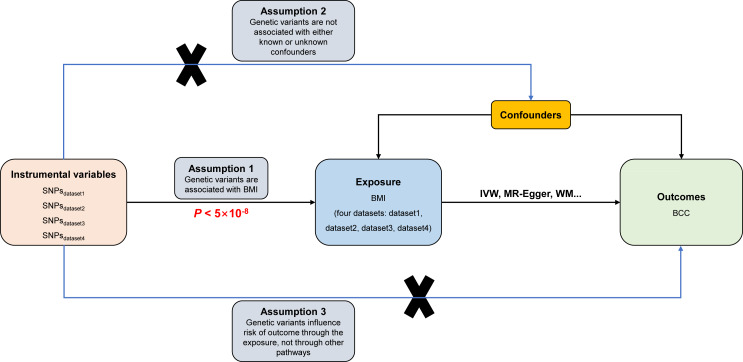
Directed acyclic graph of the MR framework investigating the causal relationship between BMI and BCC. Instrumental variable assumptions: (1) the instrument variables must be strongly associated with BMI (*P* < 5 × 10^−8^); (2) the instrument variables must not be associated with any potential confounder of BMI *vs.* BCC relationship; (3) the instrument variables should only affect the risk of BCC through BMI. SNPs, single-nucleotide polymorphisms; BMI, body mass index; BCC, basal cell carcinoma; IVW, inverse variance weighted; WM, weighted median.

In the MR research, three fundamental assumptions must be satisfied: (1) the genetic instrument variables (GIVs) must be significantly related to exposure; (2) the GIVs must not be linked to any possible exposure *vs.* outcome relationship confounders; (3) the GIVs should only impact outcome risk through exposure (Davey Smith & Hemani, 2014; Smith & Ebrahim, 2003). The assumptions and design of the MR study are shown in Fig. 1.

### Exposure data

Based on the GWAS of European ancestry, we identified 468, 282, 394 and 405 independent SNPs associated with four BMI GWASs at a genome-wide significant level (*P* < 5 ×10^−8^), respectively. SNPs’ independence was evaluated using strict criteria (*r*^2^ ≤ 0.001; clumping window, 10,000 kb). Data of all GIVs utilized were shown in Supplementary File 1. To estimate the instrument strength for each SNP in MR analyses, the F statistic was computed according to the approaches described by the previous study (Pierce, Ahsan & Vanderweele, 2011).

### Outcome data

The GWAS summary statistics data of BCC of European ancestry (GWAS ID: ebi-a-GCST90013410, 17,416 cases and 375,455 controls) were downloaded *via* the IEU Open GWAS database. Participants in the BMI research program were not screened for BCC. In other words, there is no sample overlap between BMI and BCC datasets.

### Bidirectional univariable mendelian randomization analyses

To comprehensively analyze the relationship between BMI and BCC, a bidirectional univariable two-sample MR analysis was performed for BMI and BCC as both exposure and outcome. Moreover, the inverse variance weighted (IVW) (Burgess, Butterworth & Thompson, 2013) approach was utilized as the primary causal effect estimating method to calculate the combined effect of all SNPs in this study. Simultaneously, the reliability and stability of the data were examined using the MR-Egger (Bowden, Davey Smith & Burgess, 2015), weighted median (Bowden et al., 2016), simple mode (Hartwig, Davey Smith & Bowden, 2017), and weighted mode (Hartwig, Davey Smith & Bowden, 2017) approaches. Due to variations in analysis platforms, experimental setups, inclusion populations, and SNPs, two-sample MR analysis may display heterogeneity, which could impact the assessment of causal effects. As a result, in this work, the primary IVW and MR-Egger approaches were assessed for heterogeneity. The inclusion of instrumental factors was thought to be homogeneous if the *P*-value was greater than 0.05, and the effect of heterogeneity on the assessment of causal effects was therefore disregarded. If the heterogeneity existed, then the IVW (multiplicative random effects) was employed to determine the effect size. According to the assumptions mentioned above that need to be met for MR analysis, if a GIV directly influences outcomes without affecting exposure, the MR method’s fundamentals were violated. Consequently, it is crucial to investigate whether pleiotropy exists in the causal inference between exposure and outcome. The Egger model’s intercept may be used to assess pleiotropy statistically; a divergence from 0 indicates the presence of directional pleiotropy (Burgess & Thompson, 2017). Moreover, the presence of pleiotropy in the analysis was also determined in this study using the MR-pleiotropy residual sum outlier (MR-PRESSO) (Verbanck et al., 2018). If *P* > 0.05, pleiotropy in the causal analysis was improbable, and its effects can be discounted. This study also employed the leave-one-out approach for sensitivity analysis, in which the MR was carried out again with each SNP being removed in turn (Cui & Tian, 2021). If potentially influential SNPs were found during the leave-one-out sensitivity analysis, we cautiously drew inferences. The directionality that exposure causes outcome was confirmed utilizing the MR Steiger test, *P* < 0.05 was defined as statistically significant.

### Meta-analysis

To enhance the accuracy of the genetically predicted effect of BMI on BCC, we also performed a meta-analysis that included the four BMI-associated datasets mentioned above. We conducted a single-arm meta-analysis using the ‘meta’ package of R (version 3.6.0) software (Xu et al., 2018) to estimate the average genetically predicted effect of BMI on BCC. A fixed-effects model was used to combine the ORs from several research. For the purpose of visually assessing the consequences of pooling, forest plots were established. The *I*^2^ statistic was utilized in order to evaluate the degree of heterogeneity present among the studies. A value of *I*^2^ that fell between 25 and 50 percent was regarded as representing mild heterogeneity, a value of *I*^2^ that fell between 50 and 75 percent represented moderate heterogeneity, and a value of *I*^2^ that fell greater than 75 percent represented severe heterogeneity (Higgins et al., 2003). Moreover, the chi-squared-based Q was also utilized to assessed the heterogeneity across studies.

## Results

### Bidirectional univariable MR analysis

To increase the reliability of the results, we selected four different BMI-related GWAS datasets. All 468, 282, 394, and 405 independent SNPs associated with four different BMI datasets were available in the summary statistics for BCC, respectively. The F statistic of those SNPs was greater than 10 in four BMI datasets, indicating a low risk of weak-instrument bias (Supplementary File 1).

**Figure 2:**
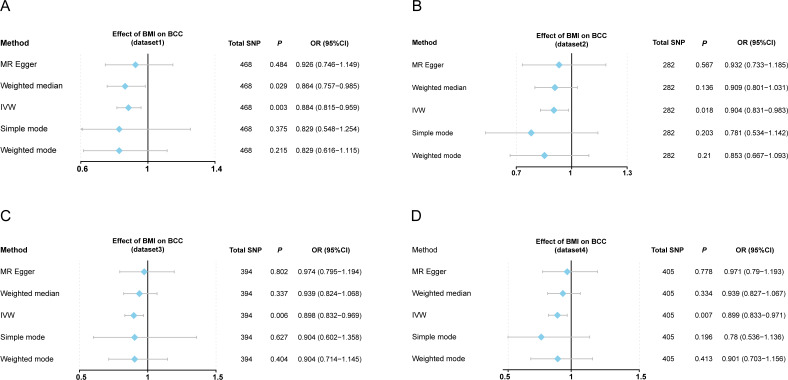
Forest plot to visualize causal effects of variation in BMI on BCC. (A–D) Effect of BMI change on BCC risk from four different BMI datasets. Presented odds ratios (OR) and confidence intervals (CI) correspond to the effects of BMI on BCC. The results of Mendelian randomization (MR) analyses using various analysis methods (MR-Egger, weighted median, IVW, simple mode, and weighted mode) are presented for comparison. Total single-nucleotide polymorphism (SNP) indicates the number of genetic variants used as instruments for MR analysis. IVW, inverse variance weighted.

**Figure 3:**
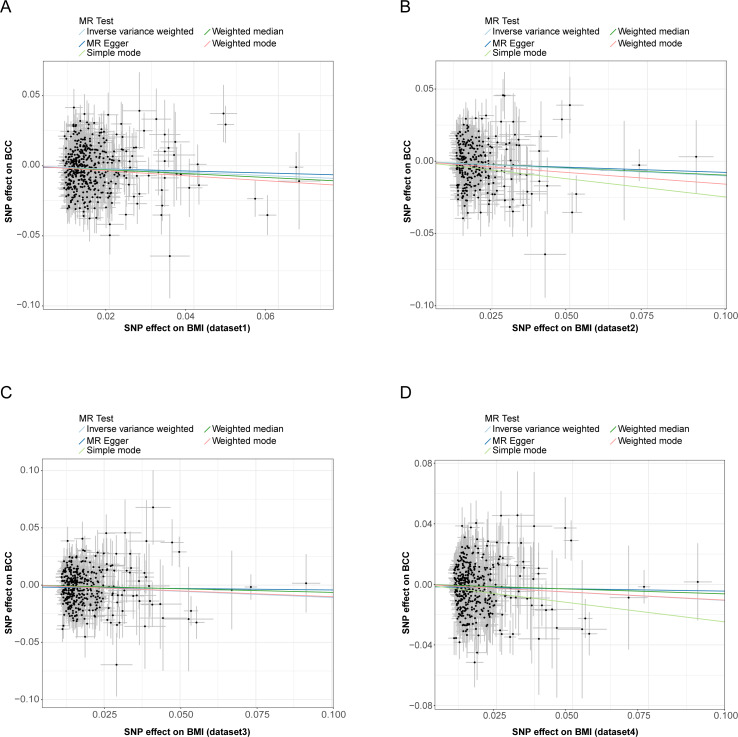
Scatter plots of BMI with the risk of BCC. (A–D) The effect of BMI-related SNP on BCC risk from four different BMI datasets. Scatter plot demonstrating the effect of each BMI-associated SNP on BCC on the log-odds scale. The slopes of each line represent the causal association for each method. MR, Mendelian randomization; SNP, single-nucleotide polymorphism; BMI, body mass index; BCC, basal cell carcinoma.

This work showed that genetically predicted BMI was inversely associated with BCC; the odds ratios were 0.884 (95% CI [0.815–0.959], *P* = 0.003), 0.904 (95% CI [0.831–0.983], *P* = 0.018), 0.898 (95% CI [0.832–0.969], *P* = 0.006), and 0.899 (95% CI [0.833–0.971], *P* = 0.007) in the IVW analysis, respectively (Table S1 and Figs. 2A–2D). Since the results of subsequent heterogeneity analysis showed substantial heterogeneity among GIVs (*P*-het < 0.05, Table S1), we used the random-effects model to estimate the above MR effect size. In addition, genetically predicted BMI was consistently associated with BCC across the different MR methods (Table S1) (Figs. 3A–3D, Fig. S1). The intercept term estimated from MR-Egger was centred at the origin (*P*-intercept > 0.05), suggesting that directional pleiotropy did not influence the results. Meanwhile, MR-PRESSO study found no outlier SNPs that increased MR pleiotropy. In addition, leave-one-out analysis showed no single SNP changed the total estimate (Fig. S2). Moreover, the SNPs explained 4.71%, 4.95%, 5.70%, and 5.76% of the variance of BMI traits, respectively. The causal assumption of BMI and BCC was verified *via* the MR Steiger test, and the result showed BMI’s influence on BCC was the correct causal direction (*P* = 0.000).

Moreover, when exposure and outcome were switched to see if reverse causation was possible, there was no indication of reverse causality from BCC to BMI (all *P* > 0.05, Fig. S3).

### Meta-analysis

**Figure 4:**
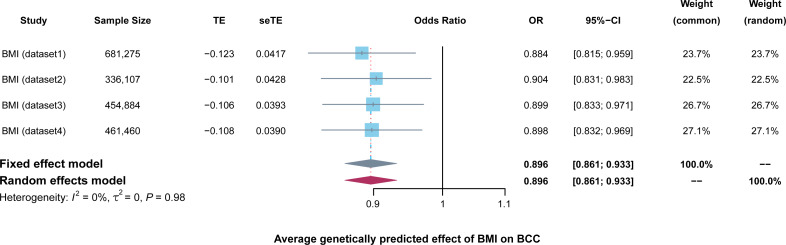
Forest plots to visualize the results of meta-analysis including four different BMI datasets. Forest plots demonstrating the average genetically predicted effect of BMI on BCC. Presented OR and CI correspond to the average effects of BMI on BCC. *I*^2^ statistic and chi-squared-based Q were utilized to assessed the heterogeneity across studies.

The results of meta-analysis were shown in Fig. 4. The results from the meta-analysis showed genetically predicted 1-SD (kg/m^2^) increase in BMI was significantly correlated with an average 10.4% decrease in the overall BCC risk; the OR was 0.896 (95% CI [0.861–0.933], *P* = 6.70E−8). Heterogeneity between the four datasets was evaluated using chi-squared-based Q and *I*^2^ tests. And the results showed there were not any heterogeneity among the four datasets (*Q* = 0.15, *I*^2^ = 0.00%, *P* = 0.98).

## Discussion

We employed MR to explore genetically predicted BMI and BCC risk and replicated findings in four independent UK Biobank populations. Using summary data from the largest BMI and BCC GWASs to date, causation was investigated using a bidirectional univariable two-sample MR, with the results evaluated further using a variety of sensitivity measures. Bidirectional univariable MR demonstrated a negative causal effect of genetically predicted BMI on BCC risk and no clear evidence for reverse causation. Moreover, further meta-analysis results also indicated a significant inverse causal effect of the average genetically predicted effect of BMI on BCC risk.

Previous studies utilizing the MR method have indicated that genetically predicted BMI was causally associated with several cancers (Liu et al., 2021; Suzuki et al., 2021; Zhou et al., 2021). To our knowledge, however, no studies have demonstrated a causal connection between BMI and BCC using MR methods. It has been shown in several earlier observational studies that BMI was linked to a lower incidence of BCC. For example, Zhang et al. (2017) reported that BMI was negatively related to early-onset BCC (OR = 0.43, 95% CI [0.26–0.71]). A prospective cohort research with 58,213 Caucasian participants revealed that the incidence of BCC decreased with rising BMI in both sexes, even after controlling for UVR exposures (Gerstenblith et al., 2012). In addition, Chan et al. (2019) found that a BMI ≥ 25 kg/m^2^ was associated with lower nonmelanoma skin cancer (NMSC) hazard rates. A prospective study on Caucasians in the US found that participants with a BMI in the obese range had a 19 percent reduced chance of getting BCC than those with a BMI in the normal range, and those with a BMI in the severely obese group had a 29 percent lower BCC risk (Pothiawala et al., 2012). These results indicated that BMI might be a protective factor for BCC. Even though they are helpful, these observational studies are susceptible to confounding factors (such as sun exposure and outdoor activities), leading to inaccurate causal inferences. For instance, after adjusting for potential confounding variables like sun exposure, Olsen et al. (2006) discovered no significant correlation between any of the anthropometric parameters (such BMI) and the incidence of BCC. Moreover, a previous study involving twin pairs indicated no clear effect of BMI on decreasing BCC risk (Milan et al., 2003). MR methods can partially address the limitations of observational studies. Our results are consistent with those of most previous observational studies, and we found that BMI has a negative effect on the risk of BCC using MR analysis. At the same time, in order to ensure the reliability of the results, we selected four independent BMI-related datasets to study the causal relationship between BMI and BCC. Importantly, the analysis results presented by these four datasets maintain a high degree of consistency. Additionally, to enhance the accuracy of the genetically predicted effect of BMI on BCC, we also performed a meta-analysis; the analysis results still showed a strong negative causal relationship between BMI and BCC.

Previous researches have not studied the mechanism between obesity (BMI over 30 kg/m^2^) and BCC; we can only draw certain assumptions. Biologically, elevated estrogen levels due to obesity (Crosbie et al., 2010) may be associated with reduced BCC risk in obese people, which has been associated with a lower risk of BCC in mouse models (Mancuso et al., 2009). In addition, Cho et al. (2008) found that estrogen might protect mice’s epidermal cytokine production and immunological function from UVR-induced (the key skin cancer risk factor) damage. These results suggest that estrogen may indeed have some protective effect against skin diseases. In addition to biological factors, the behavior of obese people also shows certain characteristics (*e.g.*, spending less time outdoors and avoiding sunbathing) (Gerstenblith et al., 2012). The observed inverse relationships between obesity and BCC risk may therefore be impacted in part by unmeasured confounding by UVR exposure.

Despite the fact that two-sample MR is an effective method for drawing causal inferences between exposures and outcomes using summary statistics, we should be prudent with our conclusions due to several limitations. First, although the leave-one-out test is a statistical method for assessing the reliance of MR results, it is more suitable for testing a good deal of SNPs rather than several SNPs. In this study, we identified a large number of SNPs from GWAS data with large samples and used these SNPs for MR analysis, thus avoiding this limitation. Second, our study was conducted utilizing populations from Europe, which limits its capacity to be generalized to a wider population. Third, it is also possible that additional factors, such as sun exposure and outdoor activities confound our results. However, it is difficult to account for the influence of other factors due to the limited availability of genetic data underpinning these traits. Finally, although our study shows that BMI negatively correlates with BCC, we still need to be cautious. Do not increase BMI excessively because the excessive increase in BMI may increase the risk of other diseases (Avgerinos et al., 2019). Therefore, in the future, we need to clarify other mediators of the causal relationship between BMI and BCC. Perhaps we can prevent BCC (the most common skin cancer in humans) by intervening with mediators to avoid side effects during the treatment of BCC by inducing an excessive increase in BMI. Of course, this requires the continuous efforts of subsequent researchers.

The study’s magnitude and the fact that BMI was measured rather than self-reported are two of its main strengths. In addition, to our knowledge, no MR has been conducted to evaluate the relationship between BMI and BCC. MR studies provided advantages over traditional observational investigations, such as reducing residual confounding risk. Additionally, we incorporated four independent BMI-related datasets to ensure the reliability of causal analysis. Critical sensitivity assessments were also undertaken to validate the assumptions of MR analyses. As a result, we were able to offer new perspectives that could aid in understanding the role of BMI in BCC incidence. In summary, using univariable MR analyses and meta-analysis, we found evidence to support BMI as a negative causal factor for BCC risk. More research is needed to determine how this possible cause-and-effect link works.

##  Supplemental Information

10.7717/peerj.14781/supp-1 Supplemental Information 1 MR density plots to visualize the overall heterogeneity of MR estimates for the effect of BMI on BCC (A–D) Represent the results of heterogeneity analysis from four different BMI datasets. MR, Mendelian randomization; SNP, single-nucleotide polymorphism. BMI, body mass index; BCC, basal cell carcinoma.

10.7717/peerj.14781/supp-2 Supplemental Information 2 Leave-one-out plots of BMI with the risk of BCC Leave-one-out analysis for IVW MR of BMI on BCC in summary-level analyses. SNP, single-nucleotide polymorphism; BMI, body mass index; BCC, basal cell carcinoma; MR, Mendelian randomization.

10.7717/peerj.14781/supp-3 Supplemental Information 3 Forest plot to visualize causal effects of variation in BCC on BMI Presented OR and CI correspond to the effects of BCC on BMI (four datasets). The results of Mendelian Randomization (MR) analyses using various analysis methods (MR-Egger, Weighted median, inverse variance weighted, Simple mode, and Weighted mode) are presented for comparison.

10.7717/peerj.14781/supp-4 Supplemental Information 4 MR Results of BMI on Risk of BCC MR, mendelian randomization; BMI, body mass index; BCC, basal cell carcinoma; IVW, inverse variance weighted; OR, odds ratio; *P*-het, *P* value for heterogeneity using Cochran Q test; *P*-intercept, *P* value for MR-Egger intercept; MR-PRESSO, Mendelian randomization-pleiotropy residual sum outlier; SNP, single-nucleotide polymorphism.

10.7717/peerj.14781/supp-5 Supplemental Information 5 SNPs associated with BMI from four datasets

10.7717/peerj.14781/supp-6 Supplemental Information 6 The STROBE-MR checklist, RAW data and R Code

## Additional Information and Declarations

### Data Availability

The following information was supplied regarding data availability: The STROBE-MR checklist, RAW data, and R Code are available in the Supplementary Files.
