# Supplementary figures and images for: Body mass index and the risk of basal cell carcinoma: evidence from Mendelian randomization analysis
Source: PeerJ. 2023 Jan 24;11:e14781. doi: 10.7717/peerj.14781 (PMC9881466; doi:10.7717/peerj.14781)

A

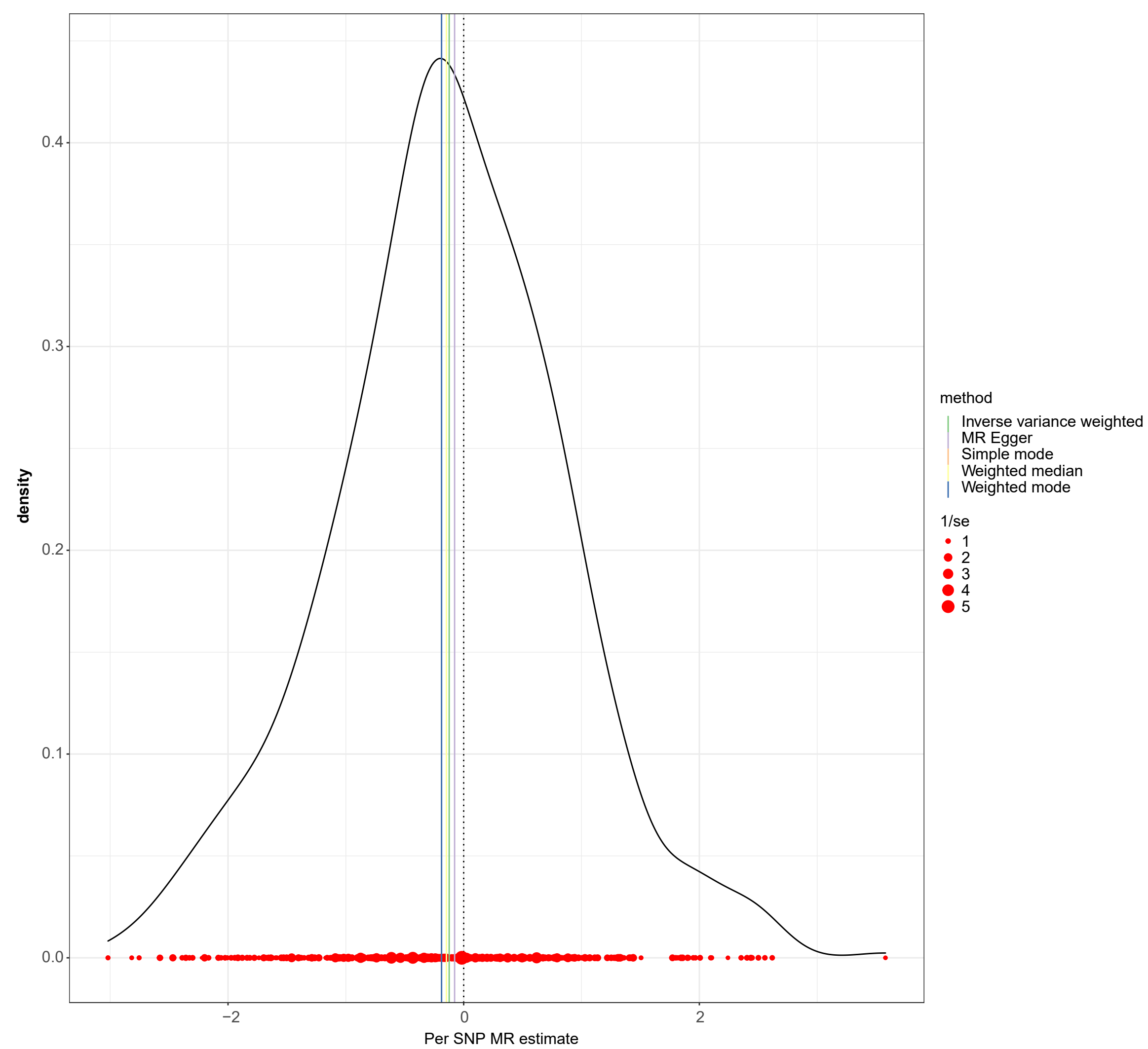

B

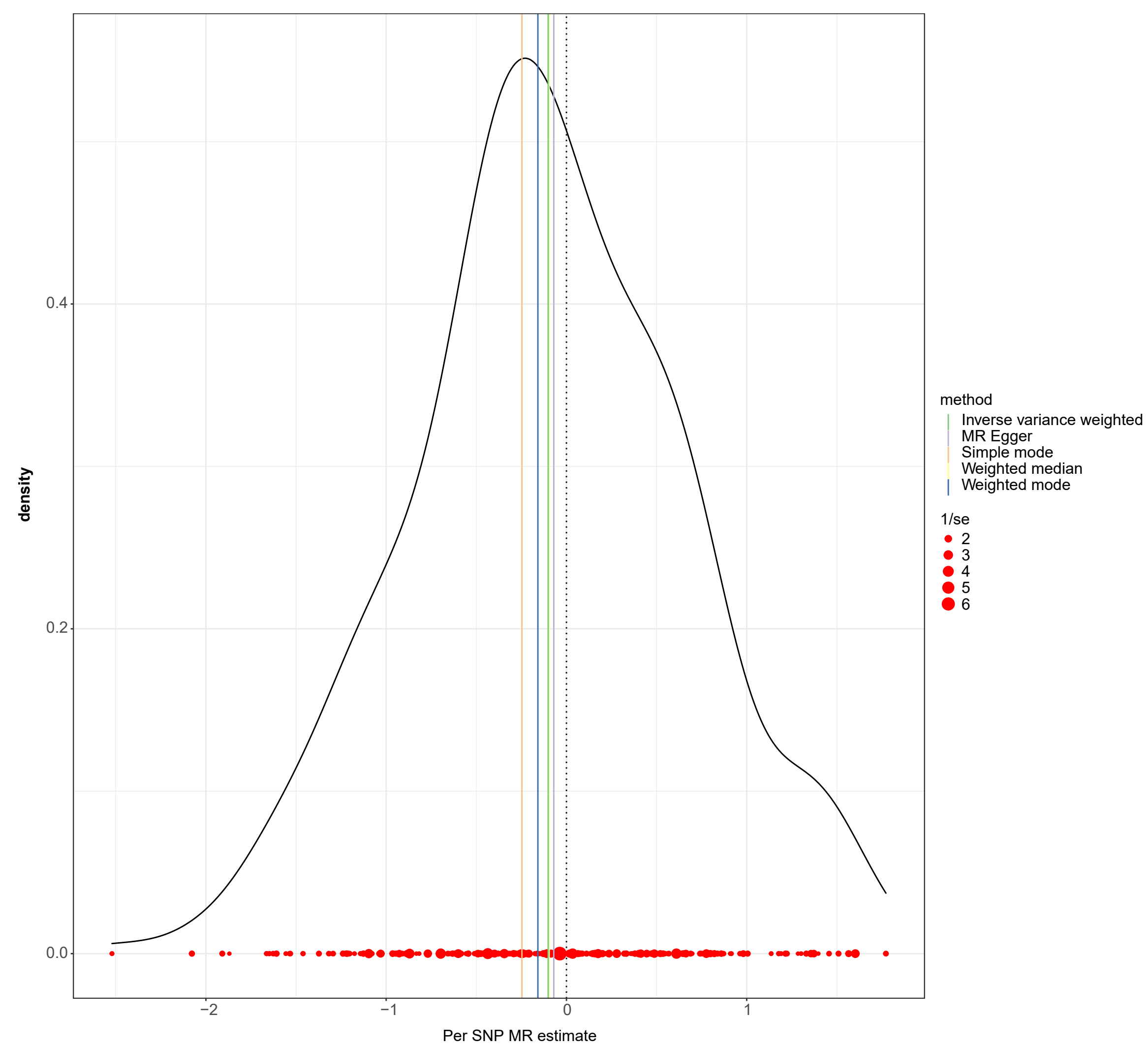

C

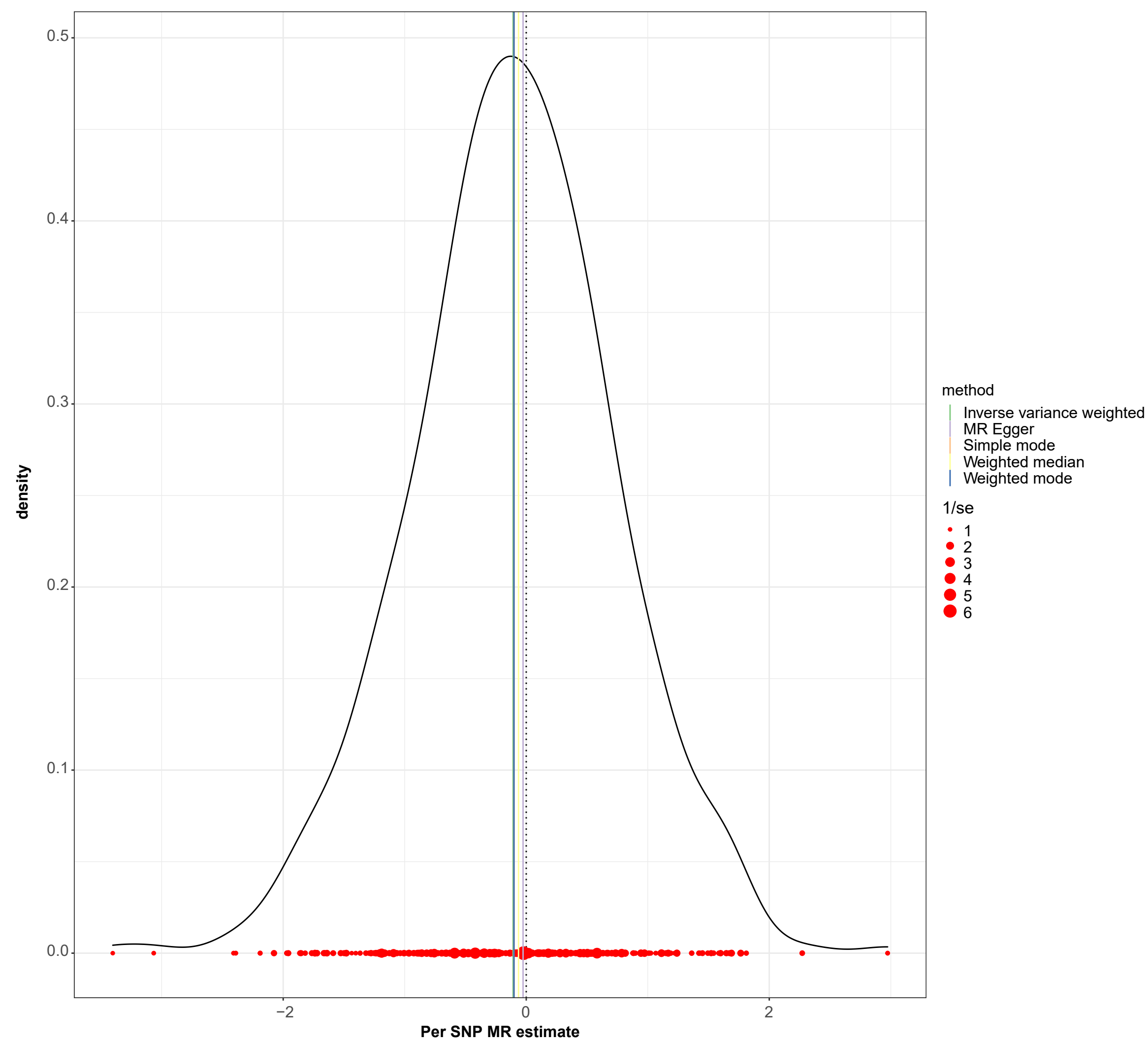

D

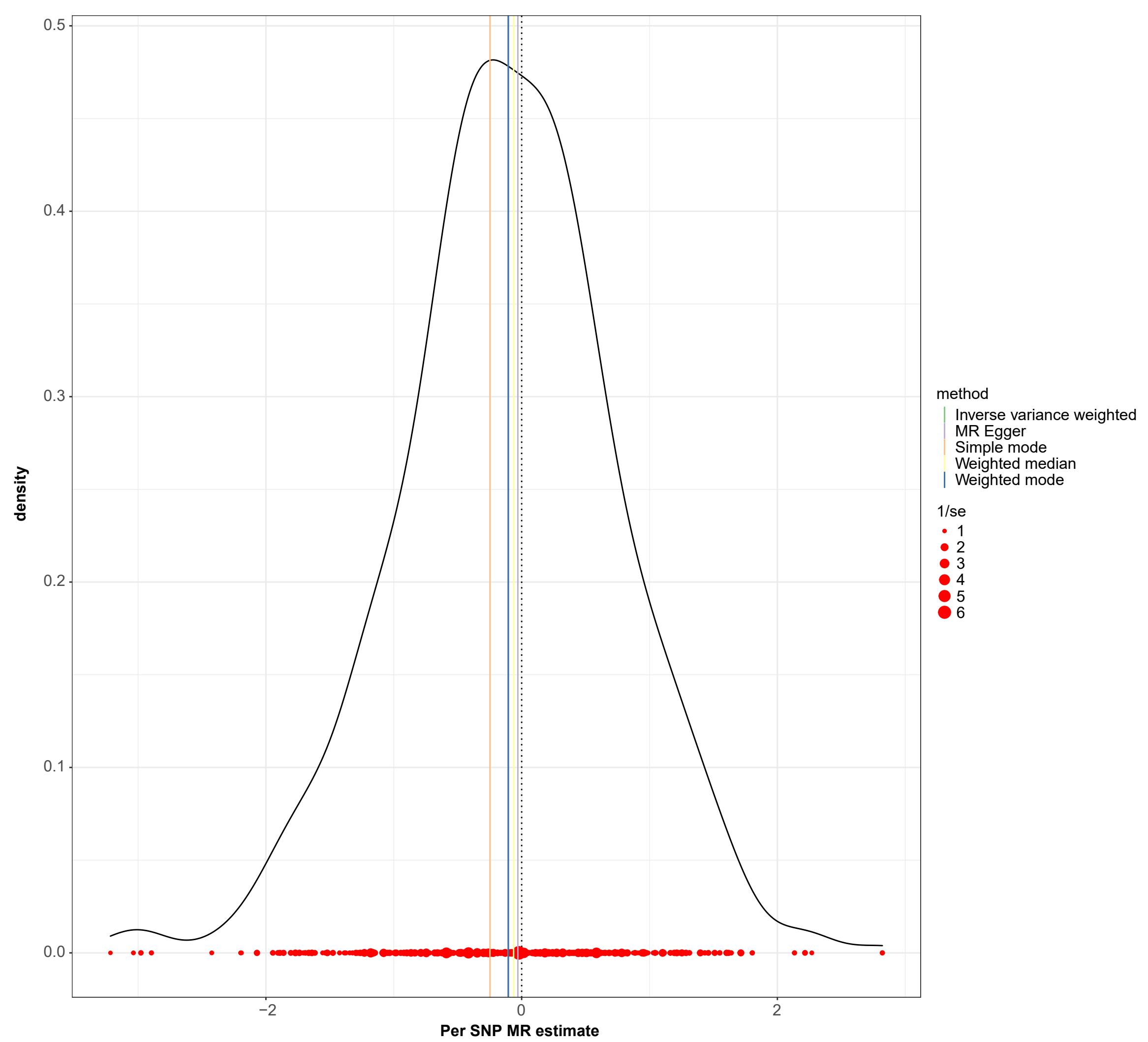

Supplement: Supplemental Information 1 — (A–D) Represent the results of heterogeneity analysis from four different BMI datasets. MR, Mendelian randomization; SNP, single-nucleotide polymorphism. BMI, body mass index; BCC, basal cell carcinoma. [file peerj-11-14781-s001.pdf]

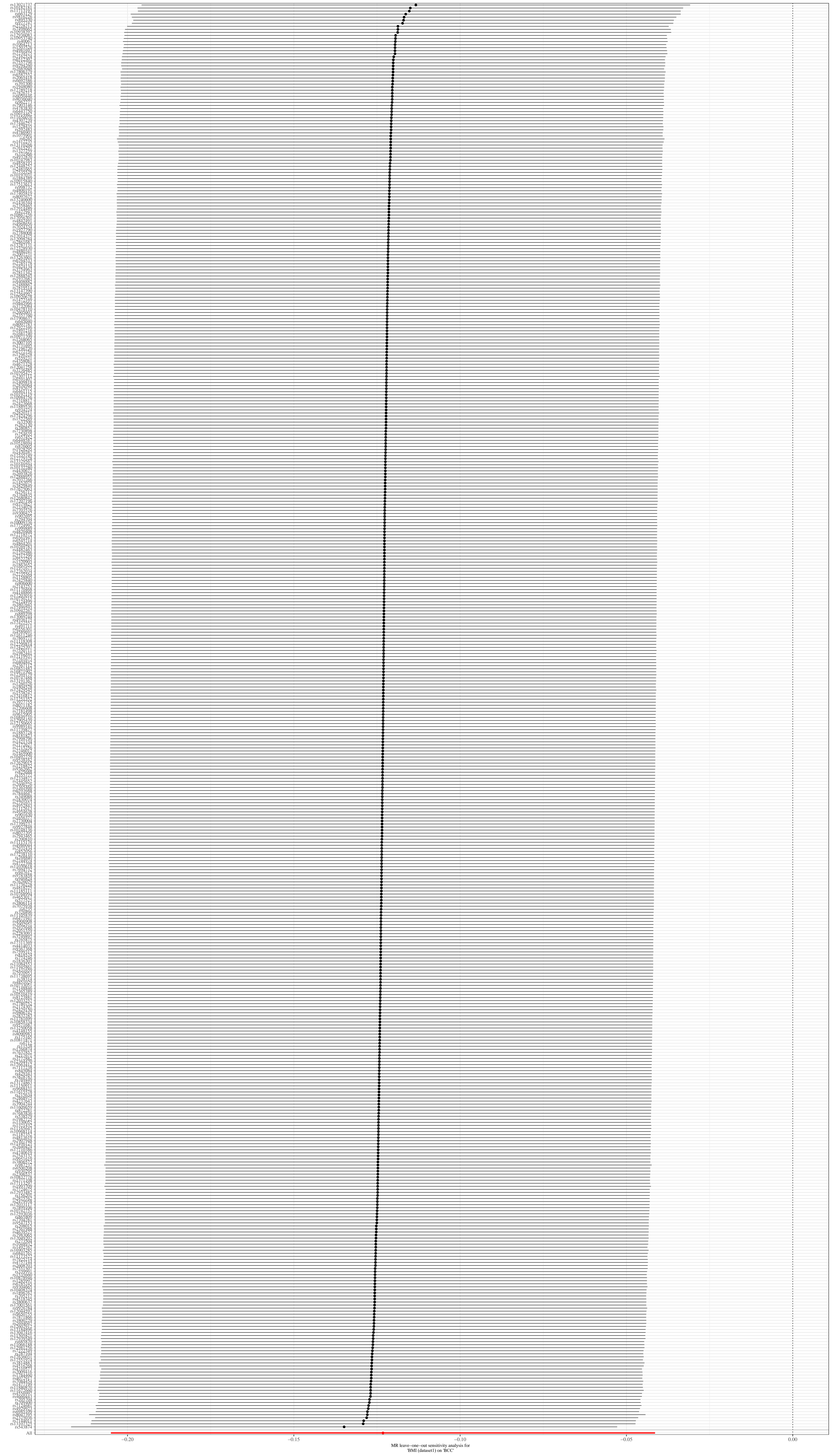

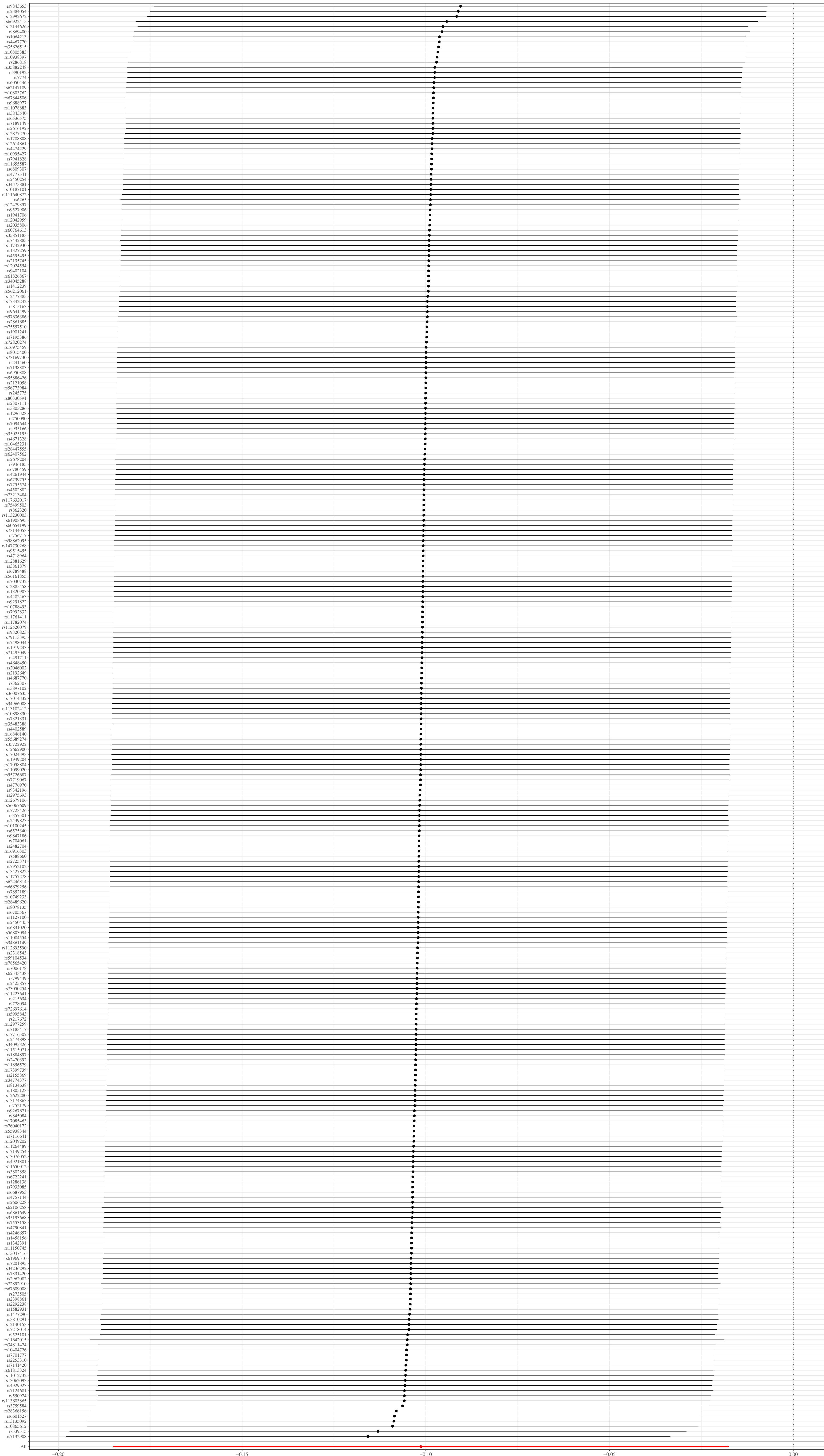

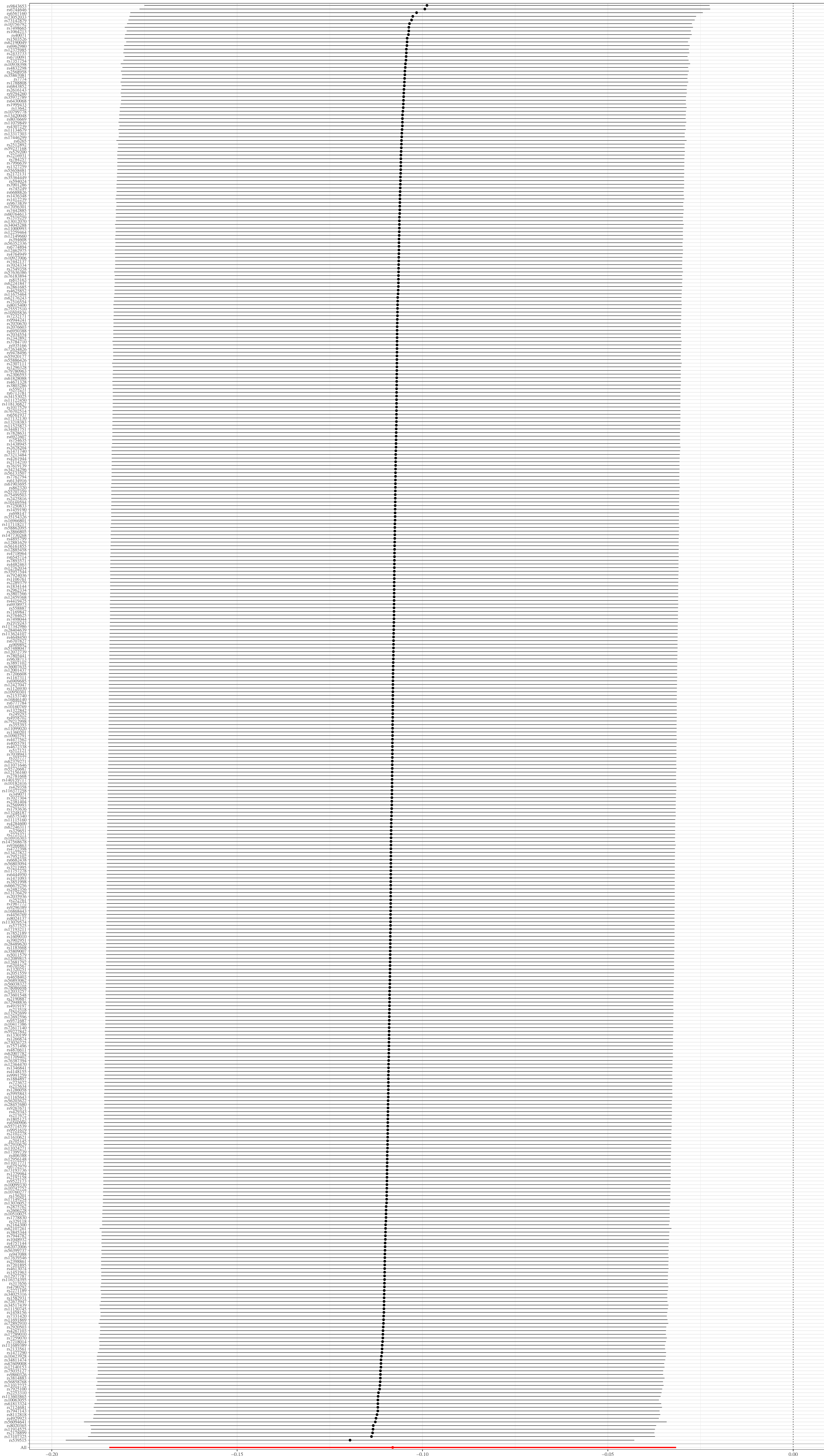

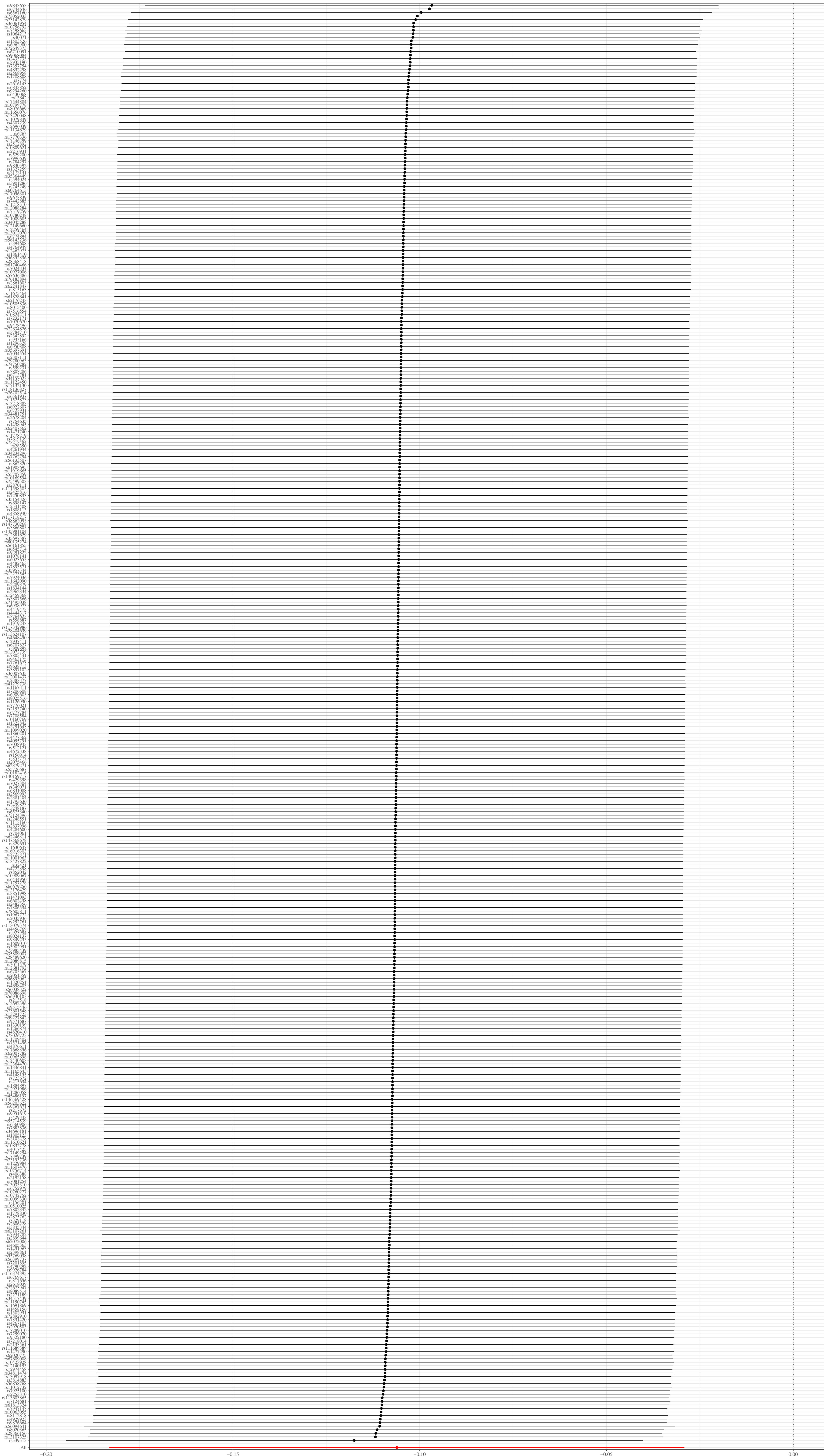

Supplement: Supplemental Information 2 — Leave-one-out analysis for IVW MR of BMI on BCC in summary-level analyses. SNP, single-nucleotide polymorphism; BMI, body mass index; BCC, basal cell carcinoma; MR, Mendelian randomization. [file peerj-11-14781-s002.pdf]

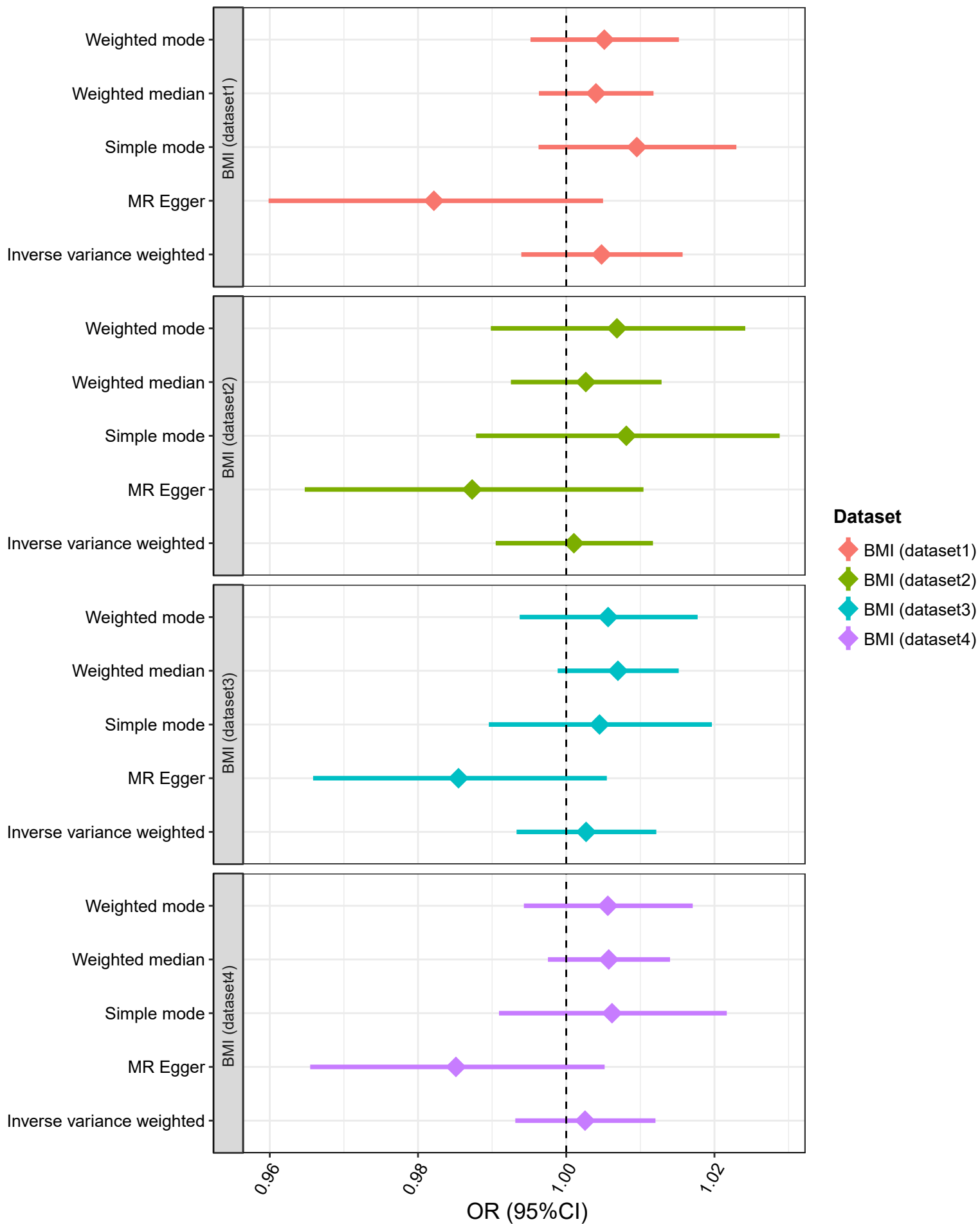

Supplement: Supplemental Information 3 — Presented OR and CI correspond to the effects of BCC on BMI (four datasets). The results of Mendelian Randomization (MR) analyses using various analysis methods (MR-Egger, Weighted median, inverse variance weighted, Simple mode, and Weighted mode) are presented for comparison. [file peerj-11-14781-s003.pdf]
