# Supplementary material for: Body mass index and the risk of basal cell carcinoma: evidence from Mendelian randomization analysis
Source: PeerJ. 2023 Jan 24;11:e14781. doi: 10.7717/peerj.14781 (PMC9881466; doi:10.7717/peerj.14781)
Supplement: Supplemental Information 4 — MR, mendelian randomization; BMI, body mass index; BCC, basal cell carcinoma; IVW, inverse variance weighted; OR, odds ratio; P-het, P value for heterogeneity using Cochran Q test; P-intercept, P value for MR-Egger intercept; MR-PRESSO, Mendelian randomization-pleiotropy residual sum outlier; SNP, single-nucleotide polymorphism. [file peerj-11-14781-s004.doc]

| **Exposure** | **Method** | **No. of SNPs** | **OR (95% CI)** | ***P*** | ***P*-het** | ***P*-intercept** |
| --- | --- | --- | --- | --- | --- | --- |
| BMI (dataset1) | MR Egger | 468 | 0.926 (0.746-1.149) | 0.484 | 1.74E-09 | 0.651 |
|  | Weighted median | 468 | 0.864 (0.757-0.985) | 0.029 |  |  |
|  | IVW | 468 | 0.884 (0.815-0.959) | 0.003 | 2.00E-09 |  |
|  | Simple mode | 468 | 0.829 (0.548-1.254) | 0.375 |  |  |
|  | Weighted mode | 468 | 0.829 (0.616-1.115) | 0.215 |  |  |
|  | MR-PRESSO (raw) | 468 | 0.877 (0.797-0.958) | 0.002 |  |  |
| BMI (dataset2) | MR Egger | 282 | 0.932 (0.733-1.185) | 0.567 | 1.17E-06 | 0.786 |
|  | Weighted median | 282 | 0.909 (0.801-1.031) | 0.136 |  |  |
|  | IVW | 282 | 0.904 (0.831-0.983) | 0.018 | 1.40E-06 |  |
|  | Simple mode | 282 | 0.781 (0.534-1.142) | 0.203 |  |  |
|  | Weighted mode | 282 | 0.853 (0.667-1.093) | 0.21 |  |  |
|  | MR-PRESSO (raw) | 282 | 0.902 (0.819-0.984) | 0.015 |  |  |
| BMI (dataset3) | MR Egger | 394 | 0.974 (0.795-1.194) | 0.802 | 6.27E-05 | 0.395 |
|  | Weighted median | 394 | 0.939 (0.824-1.068) | 0.337 |  |  |
|  | IVW | 394 | 0.898 (0.832-0.969) | 0.006 | 6.42E-05 |  |
|  | Simple mode | 394 | 0.904 (0.602-1.358) | 0.627 |  |  |
|  | Weighted mode | 394 | 0.904 (0.714-1.145) | 0.404 |  |  |
|  | MR-PRESSO (raw) | 394 | 0.893 (0.817-0.969) | 0.004 |  |  |
| BMI (dataset4) | MR Egger | 405 | 0.971 (0.790-1.193) | 0.778 | 2.05E-05 | 0.434 |
|  | Weighted median | 405 | 0.939 (0.827-1.067) | 0.334 |  |  |
|  | IVW | 405 | 0.899 (0.833-0.971) | 0.007 | 2.14E-05 |  |
|  | Simple mode | 405 | 0.780 (0.536-1.136) | 0.196 |  |  |
|  | Weighted mode | 405 | 0.901 (0.703-1.156) | 0.413 |  |  |
|  | MR-PRESSO (raw) | 405 | 0.892 (0.816-0.968) | 0.004 |  |  |
